# Selective inhibitors of nuclear export (SINE) as novel therapeutics for prostate cancer

**DOI:** 10.18632/oncotarget.2174

**Published:** 2014-07-07

**Authors:** Janet Mendonca, Anup Sharma, Hae-Soo Kim, Hans Hammers, Alan Meeker, Angelo De Marzo, Michael Carducci, Michael Kauffman, Sharon Shacham, Sushant Kachhap

**Affiliations:** ^1^ Department of Oncology, The Sidney Kimmel Comprehensive Cancer Center, Johns Hopkins Medical Institutions, Baltimore, MD; ^2^ Karyopharm Therapeutics, Natick, MA

**Keywords:** Nucleocytoplasmic transport, CRM1, XPO 1, SINE inhibitors, prostate cancer

## Abstract

Mislocalization of proteins is a common feature of cancer cells. Since localization of proteins is tightly linked to its function, cancer cells can inactivate function of a tumor suppressor protein through mislocalization. The nuclear exportin CRM1/XPO 1 is upregulated in many cancers. Targeting XPO 1 can lead to nuclear retention of cargo proteins such as p53, Foxo, and BRCA1 leading to cell cycle arrest and apoptosis. We demonstrate that selective inhibitors of nuclear export (SINE) can functionally inactivate XPO 1 in prostate cancer cells. Unlike the potent, but toxic, XPO 1 inhibitor leptomycin B, SINE inhibitors (KPT-185, KPT-330, and KPT-251) cause a decrease in XPO 1 protein level through the proteasomal pathway. Treatment of prostate cancer cells with SINE inhibitors lead to XPO 1 inhibition, as evaluated by RevGFP export assay, leading to nuclear retention of p53 and Foxo proteins, consequently, triggering apoptosis. Our data reveal that treatment with SINE inhibitors at nanomolar concentrations results in decrease in proliferation and colonogenic capacity of prostate cancer cells by triggering apoptosis without causing any cell cycle arrest. We further demonstrate that SINE inhibitors can be combined with other chemotherapeutics like doxorubicin to achieve enhanced growth inhibition of prostate cancer cells. Since SINE inhibitors offer increased bioavailability, reduced toxicity to normal cells, and are orally available they can serve as effective therapeutics against prostate cancer. In conclusion, our data reveals that nucleocytoplasmic transport in prostate cancer can be effectively targeted by SINE inhibitors.

## INTRODUCTION

Protein localization is tightly linked to its function [1, 2]. Improper localization of a nuclear protein to the cytoplasm can render it functionally inactive. Hence, spatial and temporal localization of protein molecules in the cell is tightly regulated by transporters [1, 2]. In the nucleus, protein transport is carried by a group of proteins belonging to the karyopherin family of transporters. Generally, any molecule above 42kD, a size which does not qualify for passive diffusion across the nuclear membrane barrier, is actively transported through the nuclear pore [3]. Import of protein inside the nucleus is carried by importins while export of RNA and proteins is carried by exportins [4]. Among the seven known exportins present in the mammalian cell, Exportin 1 (XPO 1, also called CRM1) is the most studied prototype [5, 6]. XPO 1 binds to leucine rich nuclear export sequences present in the cargo proteins to export them out of the nucleus [7]. However the affinity of XPO 1 alone to nuclear export sequences is low which is exponentially enhanced when bound to active RanGTPase [8, 9]. GTP bound active Ran along with XPO 1 and the cargo protein forms a ternary complex that is exported out of the nuclear pore complex. Outside the nucleus, aided by cytoplasmic RanGTPase activating protein, RanGTP undergoes GTP hydrolysis causing XPO 1 to lose its affinity for the nuclear export sequence and release the cargo in the cytoplasm [6, 10]. Normal cells utilize nuclear transporters to maintain cellular physiology and homeostasis, while cancer cells dysregulate nuclear transporters to mislocalize nuclear proteins to gain selective survival and growth advantage [4]. Hence, modulation of nucleocytoplasmic transport by small molecule modulators against cancer is actively sought.

Increased expression of XPO 1 protein has been noted in several cancer types including pancreatic [11], cervical [12], ovarian [13], mantle cell lymphoma [14], and glioma [15]. Cancer cells utilize XPO 1 to export, among others, p53, APC, p21, p27, Foxo, BRCA1, ATM, and TopoI to the cytoplasm [4, 5, 10, 16]. Restriction of these key gatekeeper and caretaker proteins to the cytoplasmic compartment prevents them from suppressing tumor growth. Since half of the cancers retain a wild type p53 gene, restoring nuclear p53 function through inhibition of XPO 1 could trigger cell cycle arrest or apoptosis [17, 18]. This makes XPO 1 an attractive target in a variety of cancers. Leptomycin B, a known potent and selective inhibitor of XPO 1, covalently binds to the Cys^528^ residue in the nuclear export signal (NES)-binding groove of XPO 1 and inactivates it [19]. Although potent, this compound suffers from being very toxic to normal cells resulting in a very narrow therapeutic window. Knowledge about overt toxicity, gained from a Phase I clinical trial, led to discontinuation of leptomycin B from further clinical development [20]. This however did not deter the search for novel compounds, with increased efficacy and reduced toxicities that could target nucleocytoplasmic transport. Selective inhibitors of nuclear transport (SINE) are novel inhibitors of XPO 1 that differ structurally from leptomycin B but like leptomycin B they covalently bind to Cys^528^ residue in the central conserved region of XPO 1 and inactivates it [14, 19, 21, 22, 23, 24]. In this study, we investigated the effect of three SINE inhibitors KPT185, KPT330, and KPT251 on prostate cancer. These compounds selectively bind to XPO 1 and inhibit its function at the nanomolar range. KPT301, the 10-fold less active trans-isomer of KPT185, was included as a negative control. Our data indicate that SINE inhibitors, unlike leptomycin B, decrease XPO 1 protein level through proteasomal degradation and selectively trigger apoptosis and inhibit prostate cancer cells but not normal prostate cells.

## RESULTS

### SINE inhibitors inhibit XPO 1 function in prostate cancer cells

**Figure 1:**
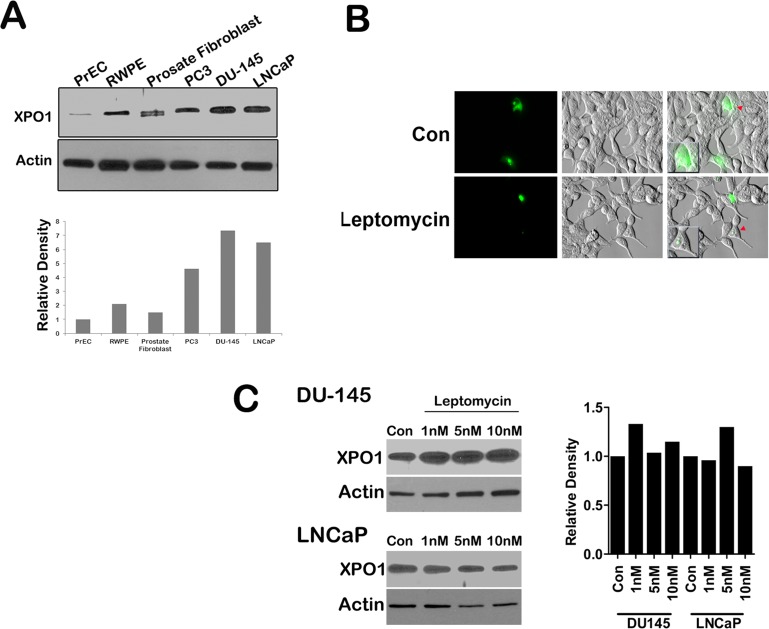
XPO 1 is upregulated and active in prostate cancer cell lines (A) Prostate cancer cells lines (PC3, DU-145, and LNCaP) show increased expression of XPO 1 protein as compared to normal prostate epithelial cells (PrEC and RWPE) as well as prostate fibroblasts. Upper panel is a representative blots and lower panel is densitometric analysis of XPO 1 bands after normalizing with housekeeper actin; (B) Prostate cancer cell lines harbor a functional XPO 1 protein as demonstrated by RevGFP export assay. LNCaP cells were transfected with the RevGFP construct, followed by treatment with leptomycin B at a final concentration of 10ng/ml and imaged at the end of 2h. Leptomycin B treated cells showed clear nuclear and nucleolar location of RevGFP as compared to untreated cells. Multiple fields per slide were imaged. Insets show magnified image of a single cell indicated by red arrow heads; (C) Protein expression of XPO1 in prostate cancer cells (DU-145 and LNCaP) do not change after treatment with leptomycin B. Cells were treated with leptomycin B at the indicated concentrations for 48h, and then subjected to immunoblot analysis using anti-XPO 1 antibody. Actin served as a loading control. Graph depicts densitometric analysis of XPO 1 bands after normalizing with housekeeper actin. A and C are representative blots of at least three independent experiments, B is a representative image of a single field from two independent experiments.

**Figure 2:**
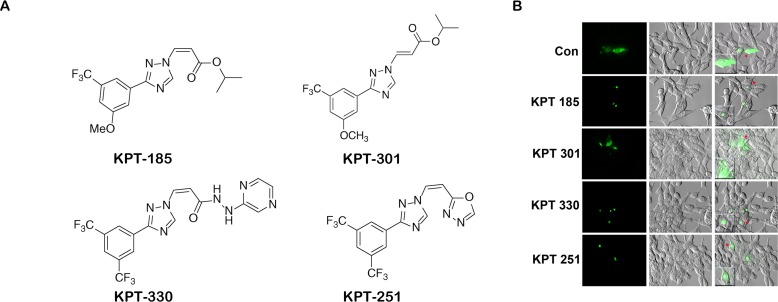
SINE inhibitors functionally inactivate XPO 1 in prostate cancer cells (A) Chemical structure of SINE inhibitors; (B) SINE inhibitors causes functional inactivation of XPO 1. LNCaP cells were transfected with RevGFP and treated with 1uM of SINE inhibitors for 2h after 24h post transfection. Multiple fields per slide were imaged. Insets show magnified image of a single cell pointed out by the red arrow heads. Figure is a representative image of a single field from two independent experiments.

Increased expression of XPO 1 protein is found in many cancer cell lines and tissues [11, 13, 14]. To investigate the expression of XPO 1 in normal prostate and prostate cancer cell lines, we conducted a western blot analysis on lysates of normal prostate epithelial cells (PrEC), prostate fibroblast, and androgen responsive (LNCaP) and non-responsive (DU-145 and PC3) prostate cancer cells. Western blots indicate that XPO 1 is overexpressed in prostate cancer cells as compared to normal prostate epithelial cells or prostate fibroblasts (Fig. 1A).To investigate whether prostate cancer cell lines harbored a functional XPO 1 protein, we utilized a green fluorescent Rev protein which localizes to the nucleus and the nucleolus. Export of Rev out of the nucleus into the cytoplasm is dependent on XPO 1 [25]. Transfection of prostate cancer cell lines with the RevGFP construct revealed the presence of GFP fluorescence in all the three cellular compartments,indicating a functional XPO 1 in prostate cancer cell lines (data not shown). To investigate whether leptomycin B is able to functionally inactivate XPO 1 in prostate cancer cells, we treated RevGFP transfected LNCaP cells with leptomycin B. This led to nuclear and nucleolar retention of RevGFP, indicating leptomycin B is able to functionally inactivate XPO 1 in LNCaP cells (Fig. 1B). Inhibition of XPO 1 with XPO 1 specific inhibitor, leptomycin B, in DU-145 and LNCaP cells demonstrated that leptomycin B treatment does not lead to a decrease in XPO 1 protein levels, inferring that XPO 1 is functionally inactivated by leptomycin B without a loss of protein expression (Fig. 1C). These data indicate that XPO 1 is functionally active in prostate cancer cells and can be specifically inhibited by leptomycin B. We next used SINE inhibitors (Fig. 2A) in prostate cancer cells and tested their ability to functionally inactivate XPO 1. Treatment of RevGFP transfected LNCaP cells with 1μM of SINE inhibitors led to nuclear and nucleolar retention of RevGFP indicating that SINE inhibitors can functionally inhibit XPO 1 in prostate cancer cells. RevGFP localization in cells treated with the trans-isomer (KPT301) was found similar to that of controls (Fig. 2B and supplementary Fig. S1). This suggested that, like leptomycin B, KPT185,-330, and -251 are potent inhibitors of XPO 1 function.

### SINE inhibitors decrease XPO 1 protein through proteasomal mediated degradation

**Figure 3:**
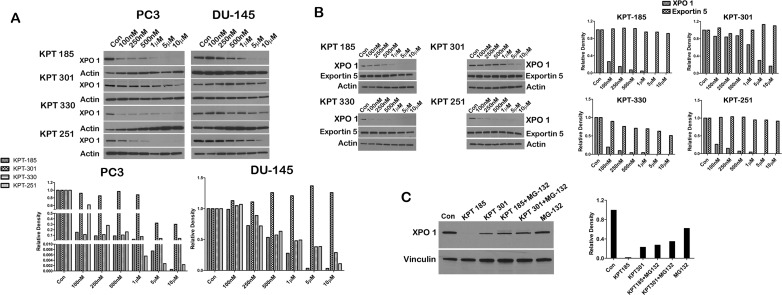
SINE inhibitors cause proteasomal degradation of XPO 1 (A) Prostate cancer cells lines (PC3 and DU-145) were treated with SINE inhibitors at the indicated concentrations for 48h, and then subjected to immunoblot analysis using anti-XPO 1 antibody. Actin served as a loading control. Graph below is a densitometric analysis of XPO 1 bands after normalizing with housekeeper actin; (B) LNCaP cells were treated with SINE inhibitors at the indicated concentrations for 48h, and then subjected to immunoblot analysis using anti-exportin5 antibody. Actin served as a loading control. Graph to the right is densitometric analysis of XPO 1 and exportin 5 bands after normalizing with housekeeper actin; (C) LNCaP cells were treated with SINE inhibitors alone at a concentration on 1μM, in combination with the proteasomal inhibitor MG-132, and with MG-132 alone at a final concentration of 10μM for 12h, and then subjected to immunoblot analysis using anti-XPO 1 antibody. Vinculin served as a loading control. Graph to the right is densitometric analysis of XPO 1 bands after normalizing with housekeeper vinculin. Images are representative blots of three independent experiments.

It is known that SINE inhibitors covalently bind to Cys^528^ residue of XPO 1 and thereby inhibit enzymatic function of XPO 1 [14, 19, 21, 22, 23, 24]. In this respect, the mode of action of the SINE inhibitors is similar to leptomycin B. However, when we treated prostate cancer cell lines with increasing concentration of SINE inhibitors we found that all the three inhibitors, except KPT301, decreased XPO 1 protein at lower nanomolar concentrations (Fig. 3A). LNCaP cells were most sensitive to XPO 1 downregulation followed by PC3 and DU-145 cells. Most strikingly, KPT301 which is 10 fold less potent exhibited a decrease in XPO 1 protein at concentrations 10 fold greater than other SINE inhibitors. In order to dismiss that this was not due to a general decrease in all exportins, we probed the lysates for a related exportin, namely exportin5. Treatment with increasing concentration of SINE inhibitors did not decrease exportin 5 protein levels indicating that downregulation was highly specific to XPO 1 (Fig. 3B). Downregulation of XPO 1 could be either due to transcriptional or post transcriptional regulation. We chose to test the likelihood that XPO 1 is post translationally decreased by SINE inhibitors. We argued that although SINE inhibitors bind to the same residue in XPO 1 as leptomycin B, it is likely that SINE inhibitors change the conformation of XPO 1 such that it is recognized by the proteasomal degradation machinery and thereby degraded. To investigate this possibility, we treated LNCaP cells with 1μM of KPT185 and its trans-isomer KPT301, as a single agent, and in combination with the proteasome inhibitor MG-132. While KPT185 as a single agent decreased XPO 1 protein levels, combination of KPT185 with MG-132 led to stabilization of XPO 1 protein indicating that decreased protein level seen after treatment with SINE inhibitors was a result of proteasomal mediated degradation (Fig. 3C).

### Inhibition of XPO 1 by SINE inhibitors leads to retention of tumor suppressors Foxo and p53 in the nucleus

**Figure 4:**
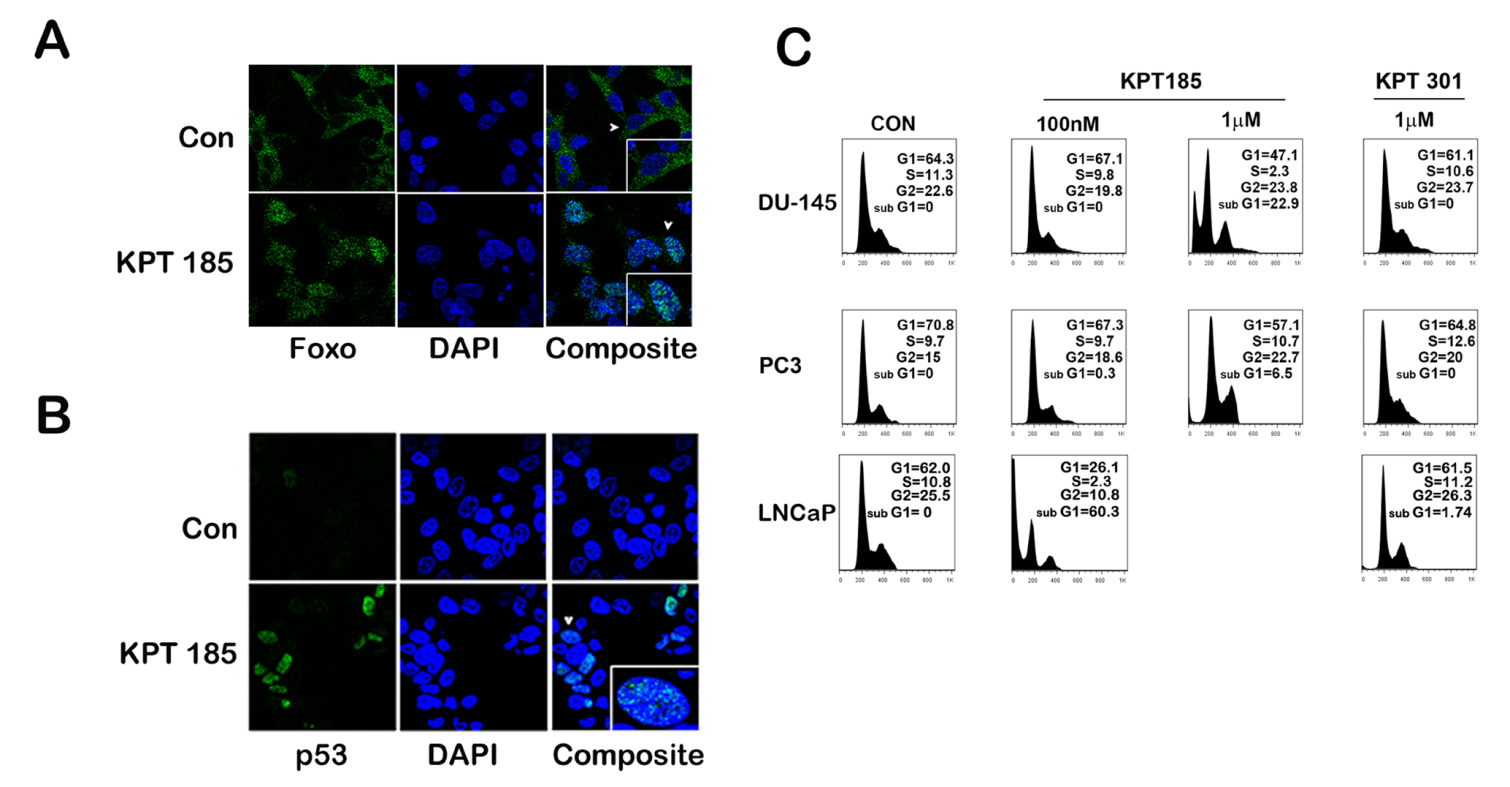
SINE inhibitors help nuclear retention of tumor suppressor proteins like p53 and FOXO by functionally inhibiting XPO 1 Representative images of cellular distribution (nucleus/cytoplasm) determined by confocal imaging. LNCaP cells were treated with SINE inhibitor KPT185 at a concentration of 1μM for 48h. Cells were fixed and stained for FOXO protein (A) and p53 (B) and detected by using secondary Alexa 488-coupled goat anti-rabbit IgG (green). Nuclei are stained with DAPI (Blue). Insets show magnified view of a single cell pointed out by the white arrow heads. (C) Quantification of the sub G0/G1 population induced by treatment with SINE inhibitors. Prostate cancer cells (DU-145, PC3, and LNCaP) were treated at the indicated concentrations. Forty-eight hours later, cells were harvested, stained with propidium iodide and analyzed by flow cytometry to quantify the sub G0/G1 population. Very few viable cells remained in LNCaP cells after treatment with 1μM of KPT185 for any meaningful analysis. A, B and C are representative images of three independent experiments.

Mislocalization of nuclear tumor suppressor protein precludes it from executing its tumor suppressor function thereby leading to an increase in tumor aggressiveness and progression. Pertaining to prostate cancer, recurring inactivation of PTEN due to mutation or deletion is a common recurrence in prostate cancer [26]. Inactivation of PTEN leads to an increase in Akt kinase activity which in turn labels the transcription factor Foxo with an inactivating phosphorylation mark [27, 28]. Phosphorylated Foxo is exported out of the nucleus in a XPO 1 dependent manner and is prevented from turning on cell cycle arrest genes [29]. Further since p53 is mutated in 50% of prostate cancers, stabilization of p53 could lead to cell cycle arrest and/or apoptosis in prostate cancer that harbor wild type p53 gene [30, 31]. Since p53 is degraded in the cytoplasm through MDM2-mediated ubiqitination, preventing nuclear export of p53 could lead to its stabilization [32]. To investigate whether treatment with SINE inhibitors lead to nuclear retention and accumulation of p53 and Foxo proteins in prostate cancer cells, we treated LNCaP cells with KPT185 and studied protein localization by confocal immunofluorescence microscopy. We chose LNCaP cells as they harbor deletion of one PTEN allele and a mutation in the other allele and also retain a wild type p53 gene [33]. While untreated cells exhibited Foxo staining primarily in the cytoplasm, KPT185 treated cells demonstrated primarily nuclear Foxo proteins, indicating Foxo is retained in the nucleus after treatment (Fig. 4A). A similar observation was noted for p53. In control cells, p53 was faintly visible in the nucleus, this is due to the fact that p53 is continuously exported out and degraded in the cytoplasm [32]. However, treatment with KPT185 increased p53 nuclear retention and stabilized p53 (Fig. 4B). Nuclear retention of FOXO proteins and stabilization of p53 could trigger cell cycle arrest or apoptosis. To evaluate whether SINE inhibitors could cause either response, we first evaluated their effect on cell cycle of prostate cancer cell lines. As shown in Fig. 4C, in our experimental conditions, we did not find any significant cell cycle changes after treatment with KPT185; however, we did find an increase in sub-G1 populations, indicative of apoptotic cells, with an increase in inhibitor concentration. KPT301 did not have much effect even when treated at 1μM. LNCaP cells which harbor a wild type p53 showed a greater increase in sub-G1 population as compared to PC3 and DU-145 cells. This data suggest that SINE compounds may trigger apoptosis in prostate cancer cells without causing any cell cycle arrest.

### SINE inhibitors induce apoptosis in prostate cancer cells

**Figure 5:**
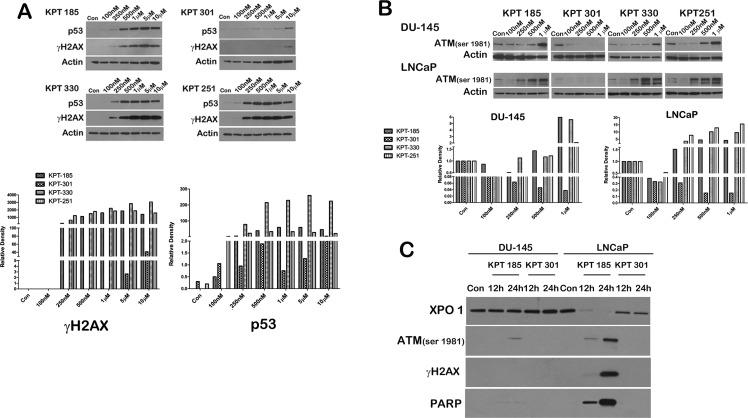
SINE inhibitors induce apoptosis in prostate cancer cells (A) SINE inhibitors stabilize p53 protein after its retention in the nucleus. LNCaP cells were treated with SINE inhibitors at the indicated concentrations for 48h. Cells were then harvested and subjected to immunoblot analysis using anti-p53 antibody. Increase in γH2AX was used to indicate an increase in double-strand breaks resulting from apoptosis. Actin served as a loading control. Graph below shows densitometric analysis of γH2AX and p53 bands after normalizing with housekeeper actin. (B) DU-145 and LNCaP cells were treated with SINE inhibitors at the indicated concentrations for 48h, and subjected to immunoblot analysis using anti-phosphoATM (Ser1981) antibody as a surrogate marker for apoptosis. Actin served as a loading control. Graph below depicts densitometric analysis of ATM bands after normalizing to housekeeper actin. (C) LNCaP cells were treated with SINE inhibitors at the indicated concentrations for either 12h or 24h. Cells were then harvested and subjected to immunoblot analysis using anti-γH2AX, anti-phosphoATM (Ser1981), and anti-cleaved PARP antibody. Images are representative blots of at least three independent experiments.

**Figure 6:**
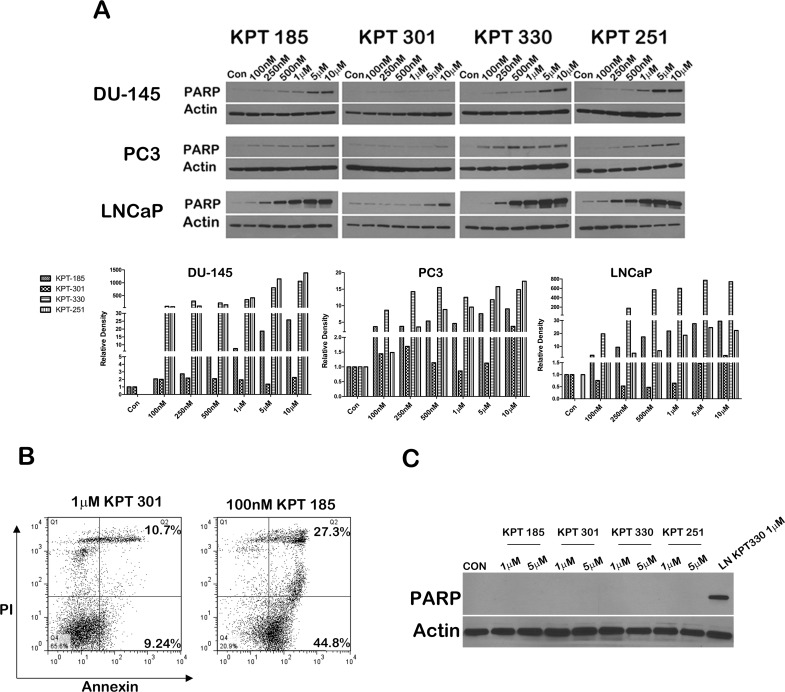
SINE inhibitors induce a dose-dependent increase in cleaved PARP (A) Prostate cancer cells lines (DU-145, PC3, and LNCaP) were treated with SINE inhibitors at the indicated concentrations for 48h, and then subjected to immunoblot analysis using anti- cleaved PARP antibody. Actin served as a loading control. Graph below is a densitometric analysis of cleaved PARP bands after normalizing with housekeeper actin; (B) Apoptosis induce by SINE inhibitors in LNCaP cells measured by flow cytometry. Cells were treated with SINE inhibitors at the indicated concentrations and stained with Annexin V and propidium iodide to quantify early versus late apoptotic population. The percentage of apoptotic cells is indicated in each case. Representative graphs of two independent experiments. (C) SINE inhibitors selectively induce apoptosis in prostate cancer cells. Prostate fibroblasts were treated with 1μM and 5μM of inhibitors for 48h and subjected to immunoblot analysis using anti-cleaved PARP antibody. LNCaP cells treated with 1M of KPT330 were used as a comparison for PARP cleavage. Actin served as a loading control. Blot images are representative blots of at least three independent experiments.

We next evaluated whether SINE inhibitors can indeed trigger apoptosis in prostate cancer cell lines. We first confirmed that p53 is indeed stabilized after treatment with SINE inhibitors by probing inhibitor treated LNCaP cell lysates for p53. As depicted in Figure 5A, whereas control lysates and KPT301 do not stabilize appreciable levels of p53, increasing doses of KPT185, -330 and -251 stabilizes p53 levels in LNCaP cells. Thus SINE inhibitors can lead to nuclear retention and stabilization of key tumor suppressors in prostate cancer cells. To evaluate apoptosis, we probed for γ-H2AX and phosphorylated ATM as surrogate markers of apoptosis [34]. As seen in Figure 5A treatment with SINE inhibitors in LNCaP cells results in stabilization of p53 protein with a concomitant increase in γ-H2AX, indicative of double strand DNA breaks resulting from apoptotic DNA fragments [34]. This data was supported by a similar increase in phosphorylated ATM in both LNCaP and DU-145 cells (Fig. 5B). We further probed the lysates for cleaved PARP, a classical marker for apoptosis. PARP was cleaved as early as 12h after treatment in LNCaP cells (Fig. 5C), perhaps indicating that LNCaP cells are slightly more sensitive to SINE inhibitors than DU-145 and PC3 cells. Nonetheless, all the prostate cancer cell lines exhibited a dose dependent increase in cleaved PARP upon treatment (Fig. 6A). We quantified apoptosis in LNCaP cells using flow cytometry. LNCaP cells were treated with KPT185 (100nM) and KPT301 (1μM) for 24h and stained for Annexin V. As shown in Figure 6B, KPT185 induced early and late apoptosis in significant population of cells as compared to trans-isomer KPT301.To determine whether apoptosis is not due to general toxicity, we treated prostate fibroblasts with SINE inhibitors at various concentrations (1μM and 5μM). These concentrations are five-fold higher than the concentrations needed to trigger PARP cleavage in prostate cancer cell lines. As seen in Figure 6C, treatment with SINE inhibitors did not trigger PARP cleavage in prostate fibroblast, indicating that SINE inhibitors cause selective death of prostate cancer cells. This data indicates that prostate cancer cells are sensitive to XPO 1 inhibition and respond to inhibition by apoptosis.

### SINE inhibitors decrease cell proliferation and clonogenic survival of prostate cancer cells

**Figure 7:**
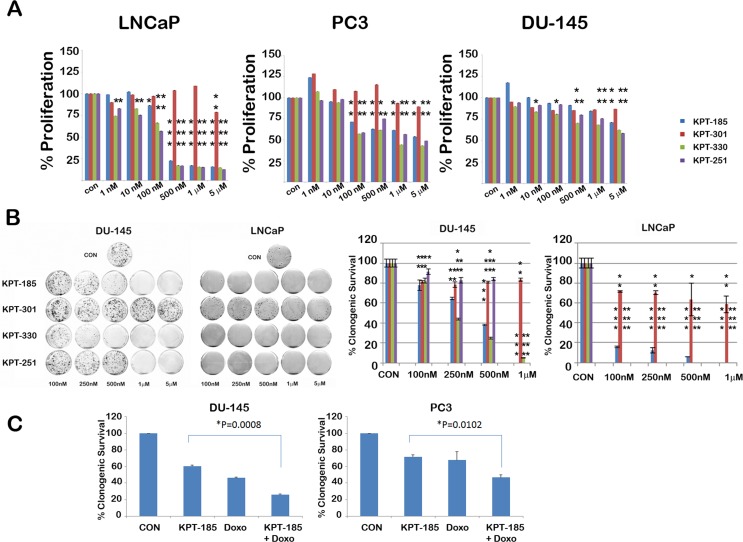
SINE inhibitors cause a decrease in cell proliferation and survival of prostate cancer cells SINE inhibitors decrease cell proliferation (A) and clonogenic survival (B) of prostate cancer cell lines in a dose-dependent manner; LNCaP cells being the most sensitive. In B, left panel shows image of clonogenic assay dishes and right graph show quantitation of percentage of clonogenic cell survival. In both A and B, cells were treated with the indicated concentrations of SINE inhibitors for 48h. Asterisks over the bars indicate significant (p<0.05, at least) statistical comparisons by the paired Student's t test. Single asterisk indicates p<0.01, double asterisk indicates p<0.001 and triple asterisks indicate p<0.0001; (C) Enhanced decrease in cell survival observed in combination treatments with KPT185 (250nM) with doxorubicin (1nM) in DU-145 and PC3 cells. Error bars represent mean ± SD (n=3). P value indicates statistical comparisons by the paired Student's t test.

To determine whether treatment with SINE inhibitors results in any meaningful decrease in proliferation and survival of prostate cancer cells, we treated them with varying doses of SINE inhibitors. Proliferation was assessed using a soluble MTS assay. As shown in Figure 7A, SINE inhibitors caused a dose dependent decrease in proliferation of all the prostate cancer lines. LNCaP cells were found to be most affected by XPO 1 inhibition as compared to the other two prostate cancer cell lines. Corroborating our data on PARP cleavage, proliferation of prostate fibroblasts was unaffected upon treatment by SINE inhibitors (Supplementary Fig. S2A). We also tested whether normal immortalized 957E/hTERT prostate epithelial cells were inhibited by SINE inhibitors. We found that only higher micormolar amounts of SINE inhibitors affected proliferation of 957E/hTERT prostate cells (Supplementary Fig. S2B). Since MTS assay is a short term assay for proliferation and does not effectively convey cell survival, we performed a cell survival clonogenic assay to evaluate the long term effect of XPO 1 inhibition. All the prostate cancer cell lines demonstrated a decrease in clonogenic capacity after treatment, LNCaP being the most sensitive of the three (Fig. 7B and Supplementary figure S3). This data clearly demonstrates that XPO 1 inhibition affects growth and survival of prostate cancer cells and that XPO 1 is a druggable target for prostate cancer treatment. Whether XPO 1 inhibition can be combined with existing therapies/drugs to achieve a greater decrease in cell growth was the next question we tried to address. Prostate cancer cell lines DU-145 and PC3 were treated with a combination of doxorubicin and KPT185 and subjected to clonogenic assays. While single agents decreased clonogenic survival as expected, combination of KPT185 with doxorubicin resulted in enhanced decrease in clonogenic potential in both the cell lines (Fig. 7C). This suggests that combination of SINE inhibitors with existing therapies is a viable and perhaps more effective option in decreasing prostate cancer growth.

## DISCUSSION

Targeted therapy that relies on pathway profile of an individual tumor is a step towards personalized medicine. Tailoring therapy against proteins which are hyperactive in tumors and essential for tumor survival can lead to better therapy. XPO 1 transports nearly 300 different cargo proteins across the nuclear envelope [4, 10, 35]. Several key pathways that fuel prostate cancer growth and survival are regulated by proteins that are cargos of XPO 1. For some of the cancer relevant cargos like p53, XPO 1 is the sole exportin [4, 10]. Since nearly half of the prostate cancers retain a wildtype p53 gene, restoring nuclear p53 function through inhibition of XPO 1 could trigger cell cycle arrest or apoptosis. This makes XPO 1 an attractive target for prostate cancer. Inhibiting prostate cancer nuclear export, independent of androgen receptor status, through agents that offer increased efficacy and reduced toxicity may benefit a bigger cohort of prostate cancer patients including castration resistant patients. Although leptomycin B is a potent and selective inhibitor of XPO 1, it suffered from being very toxic to normal cells resulting in a very narrow therapeutic window and was discontinued from further clinical development [20]. However, leptomycin B provided the prospect that XPO 1, and various cancer relevant pathways that it impacts, can be druggable. This provided the rationale and impetus for developing novel SINE compounds with reduced toxicities that potently inhibit XPO 1. Our data indicates that SINE inhibitors are effective in inhibiting XPO 1 in prostate cancer cells and may offer reduced toxicities over leptomycin B. SINE inhibitors differ structurally from leptomycin B, but like leptomycin B they covalently bind and occupy the NES-binding region of XPO 1 and inactivate it [14, 19, 21, 22, 23, 24]. These inhibitors offer increased bioavailability, reduced toxicity to normal cells, and are water soluble making them orally available [14, 22, 23]. SINE compounds have been experimentally shown to increase overall survival in the Eμ-TCL1-SCID mouse model of chronic lymphocytic leukemia with minimal weight loss or other toxicities [24]. We show that, similar to leptomycin B, SINE inhibitors can functionally inactivate XPO 1. Although both leptomycin B and SINE inhibitors bind covalently to Cys^528^ residue of XPO 1 in the NES-binding groove, unlike leptomycin B, SINE inhibitors can cause proteasomal degradation of XPO 1. This may be due to differences in their binding ability and occupancy of the NES groove as revealed by a recent X-ray crystallographic analysis [36]. Interestingly, the same study demonstrated that leptomycin B is totally reversible while SINE is slowly reversible which can explain, at least in part, why SINE inhibitors are better tolerated. Such differences may also cause sustained changes in the conformation of XPO 1 which could be recognized as a signal for degradation by the proteasomal degradation machinery.

Our data demonstrates that SINE inhibitors can lead to retention of Foxo in the nucleus, contributing to apoptosis. Additionally, our data indicates that p53 is stabilized through nuclear retention when prostate cancer cells are treated with SINE inhibitors. This could potentiate apoptosis in prostate cancer cells that have wild type p53 and could be one of the reasons why LNCaP cells that have wild type p53, as well as a hyperactive PI3K/Akt pathway, show increased sensitivity to SINE inhibitors as compared to DU-145 and PC3 cells. However, the observation that DU-145 and PC3 cells do succumb to SINE inhibitor treatment, albeit at higher nanomolar concentration compared to LNCaP cells, indicate that apoptosis is triggered in these prostate cancer cells independent of p53. Given that XPO 1 can export nearly 300 protein cargos involved in DNA repair, cell cycle regulation, and cell proliferation, death in prostate cancer cells may be triggered by a general breakdown of the nuclear export machinery. In this case cells that harbor a wild type p53 (such as LNCaP), may be poised to efficiently trigger apoptosis which may explain the increased sensitivity. Another interesting observation from our results is the sensitivity of prostate cancer cells to XPO 1 inhibition as compared to normal prostate fibroblast and epithelial cells. It is likely that cancer cells rely heavily on XPO 1 for nuclear export of deleterious nuclear tumor suppressors that can otherwise cause cell cycle arrest or apoptosis. This could be a reason why many cancer cells have an upregulation of XPO 1 protein. It is also likely that the higher proliferation and metabolic demand of cancer cells renders nuclear export obligatory for survival. Our study provides the rationale for investigating SINE inhibitors in preclinical animal models of prostate cancer for subsequent clinical translation. In the past, we and others have successfully combined various anti-cancerous agents and treatment modalities in pre-clinical settings against prostate cancer for a better therapeutic outcome [37, 38, 39]. Synergistic combinations offer better therapeutic outcomes with the added advantage of reduced toxicities against normal body cells. Our data suggests that SINE inhibitors can be combined with doxorubicin to achieve enhanced decrease in clonogenic potential of prostate cancer cells. In conclusion, our study identifies XPO 1 as a novel target in prostate cancer and demonstrates that SINE inhibitors can act as potent anti-cancerous agents.

## MATERIAL AND METHODS

### Cell lines

Prostate cancer cell lines (DU-145, PC3, and LNCaP) were obtained from ATCC, normal prostate epithelial cells (PrEC) were obtained from Lonza, HPV immortalized normal prostate epithelial RWPE cells were obtained from ATCC, and hTERT immortalized normal prostate epithelial cells (957E/hTERT) were a kind gift from Dr. John Isaacs. Human prostate fibroblasts, kindly provided by Dr. John Isaacs, were obtained from a prostate biopsy on a 62-year old patient with prostate cancer having a Gleason score of 4. All cancer cell lines and prostate fibroblast were cultured in RPMI 1640 (Corning) media supplemented with 10% FBS (Gemini Bio-Products), PrEC and 957E/hTERT cells were grown in keratinocyte serum free media (Invitrogen). Cells were grown in a humidified incubator at 37°C in a 5% CO2 atmosphere.

### Inhibitors and reagents

SINE inhibitors (KPT185, KPT301, KPT330 and KPT251) were provided by Karyopharm Therapeutics (Natick, MA). Inhibitors were dissolved in DMSO at a stock concentration of 10mM and diluted in RPMI 1640 medium at the required concentration just before treatment. Leptomycin B, MG-132 and crystal violet solution were purchased from Sigma Aldrich (St. Louis, MO). CellTiter 96™ AQueous Non-Radioactive Cell Proliferation Assay reagent was purchased from Promega (Madison, WI). RevGFP construct was kindly provided by Dr. George N. Pavlakis (NCI).

### Western blot analysis

Western blot analyses were performed as follows. Cells were plated in 100mm dishes and treated at a confluency of 50-70%. SINE inhibitors and leptomycin B were used at the specified concentrations. Treatments were carried out for 12, 24, and 48h, as specified. Post treatment, total protein was isolated and ten micrograms were used for electrophoresis and blotted on PVDF membrane. Primary antibodies were diluted in blocking buffer (either 5% milk or 5% BSA as per antibody specifications) to a 1:1000 dilution. Secondary antibodies for housekeeping proteins such as vinculin and actin, used as internal controls, were diluted at 1:4000. Blots were developed using ECL (GE Healthcare) or Femto (Pierce Biotechnology). Primary antibodies were purchased from the following source; XPO 1 (Santacruz), p53 (Calbiochem), cleaved PARP (Cell Signaling Technologies), phospho ATM (Rockland), Exportin5 (Epitomics), phospho H2AX (Millipore).

### Induction of apoptosis and determination of cell survival by flow cytometric assay

Cells were plated in 60mm dishes and treated with SINE inhibitors at a confluency of 50-70%. After 48h, both floating and attached cells were collected, washed in PBS, centrifuged, and resuspended in a fixative solution containing 10% neutral buffered saline (NBF). Cells were permeabilized in 90% methanol and stained with antibody against annexin V (Cell Signaling Technologies). Nuclei were stained with propidium iodide in PBS containing 10% FBS. Flow cytometry was performed on the FACS Calibur (BD Biosciences, San Jose, CA, USA) and data were analyzed using FlowJo software.

### Confocal and Fluorescence microscopy

Cells were plated and treated with the inhibitors at a confluency 50-70%. For the RevGFP localization experiment, cells were plated in 60mm dishes and allowed to reach a confluency of 90% before being transfected with the RevGFP construct with Lipofectamine 2000 (Invitrogen). Cells were followed and imaged at the end of 2h. Multiple fields per slide were photographed with a Nikon ECLIPSE Ti inverted research microscope (Nikon Instruments, Linthicum, MD, USA). For nuclear localization of p53 and FOXO protein, LNCaP cells were treated at 50-70% confluency. 48h post-treatment, cells were fixed in formalin and permeabilized with 0.125% Triton X 100 for 20 min at 37°C. The permeabilized cells were incubated with 5% BSA in PBS for 2h followed by overnight incubation with primary antibodies for either p53 or FOXO (Cell Signaling Technologies). Cells were washed three times with 5% BSA in PBS and probed with Alexa Fluor conjugated secondary antibodies (Invitrogen). Nuclei were counterstained with DAPI (4',6-diamidino-2-phenylindole) (Sigma Aldrich) and cells were mounted on slides. Confocal z-stack images were imaged using a Zeiss LSM 510 meta-confocal microscope (Carl Zeiss, Thornwood, NY, USA).

### Proliferation assay

Cells were plated with 100 μl complete RPMI in 96-well plates. They were allowed to adhere overnight and reach a confluency of 70% before treatment. 24, 48 and 72h post inhibitor treatment, cell viability was measured using the CellTiter 96™ AQueous Non-Radioactive Cell Proliferation Assay (Promega, Madison, WI, USA) according to the manufacturer's instructions. Absorption at 490nm was determined using a microplate reader (Molecular Devices, Sunnyvale, CA, USA).

### Clonogenic assay

Cells were plated in 60mm dishes and treated with the inhibitors at 50-60% confluency. After 48h of treatment with inhibitors, 1×10^3^ (DU-145/PC3) or 2×10^3^ cells (LNCaP) from each treated or control dish were plated in triplicate in 60mm dishes and incubated for 12 days. Colonies were stained with a crystal violet solution (Sigma Aldrich) and counted manually. Comparisons were performed using student's t-test.

## SUPPLEMENTARY FIGURES


