# Supplementary material for: Selective inhibitors of nuclear export (SINE) as novel therapeutics for prostate cancer
Source: Oncotarget. 2014 Jul 7;5(15):6102–12. doi: 10.18632/oncotarget.2174 (PMC4171616; doi:10.18632/oncotarget.2174)
Supplement: Supplementary file 1 [file oncotarget-05-6102-s001.docx]

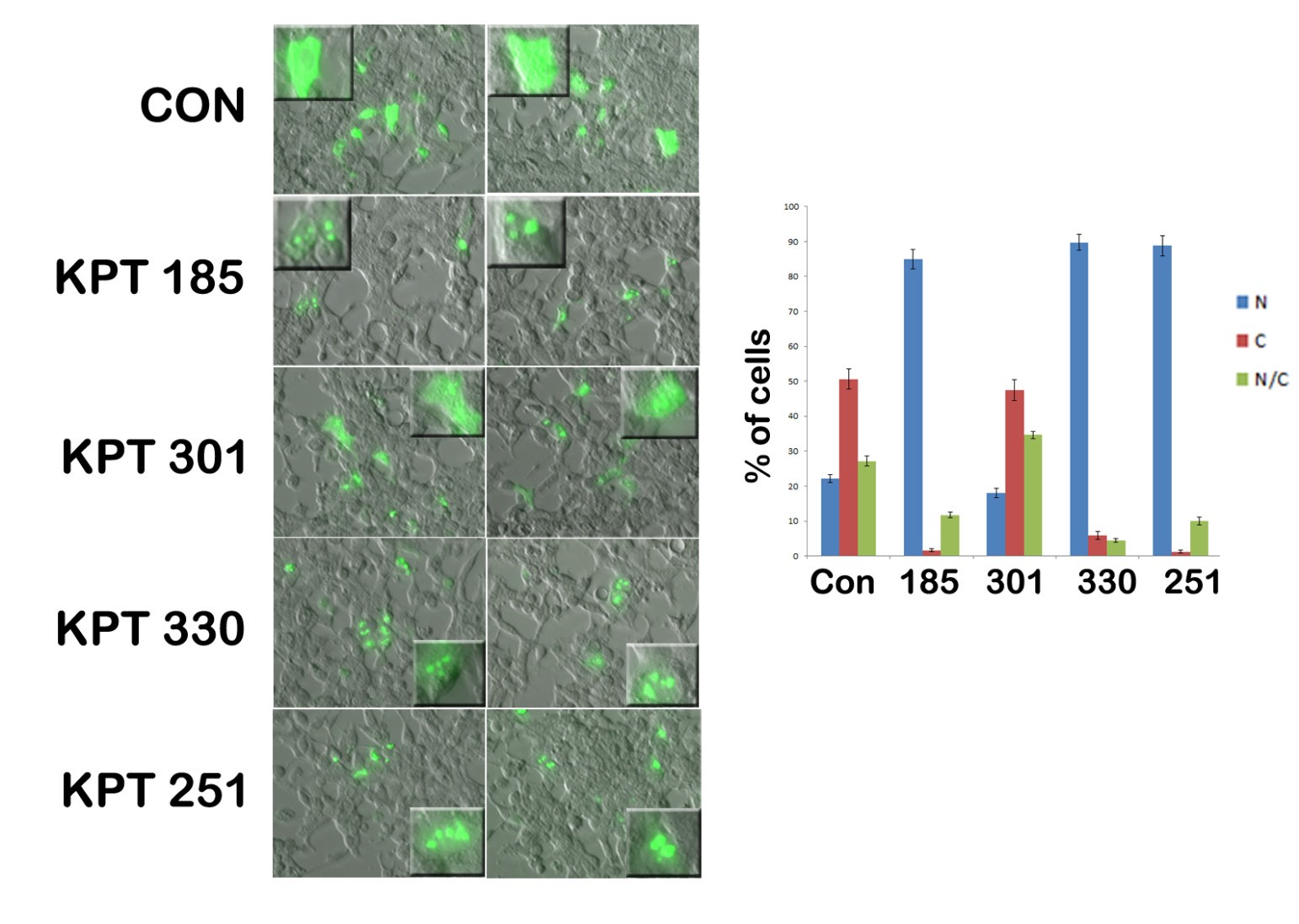


**Figure S1: XPO 1 is functionally inhibited by SINE inhibitors.** RevGFP export assay in LNCaP cells with all four SINE compounds. Cells were transfected with RevGFP reporter construct and treated with 1uM SINE inhibitors for 2h 24h post-transfection.Multiple fields per slide were imaged and RevGFP localization was measured as cytoplasmic, nuclear or both. Two fields of the multiple fields imaged are shown per treatment. Insets show magnified image of a single cell. Graph depicts percentage of cells that retain RevGFP inside the nucleus (N), or transported into the cytoplasm (C) or present both present in both nuclear and cytoplasmic compartments (N/C). Error bars represent mean ± SD (n=3).


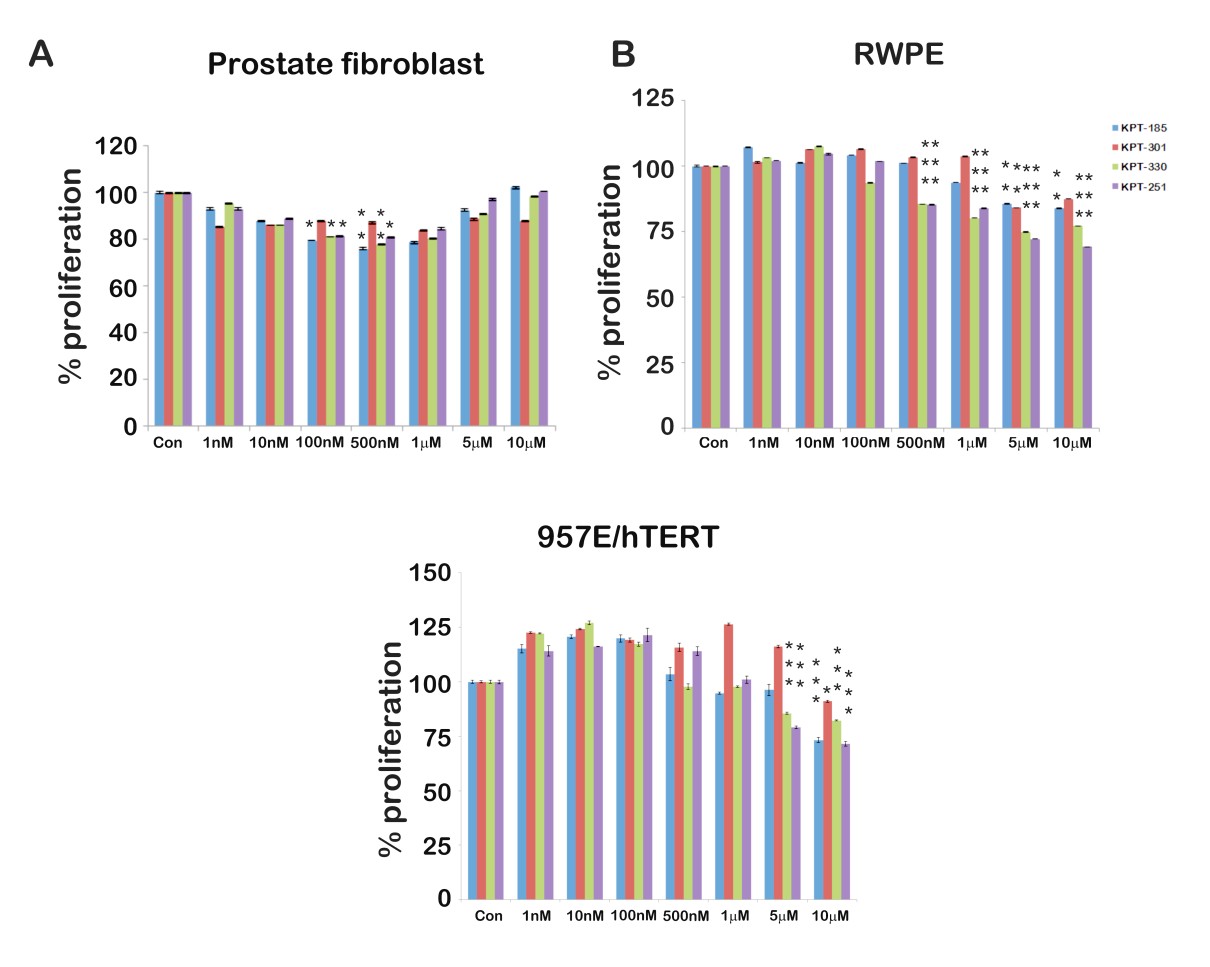


**Figure S2: SINE inhibitors do not affect in cell proliferation of normal prostate cells.**

A) Prostate fibroblasts and B) normal immortalized prostate cells 957/EhTERT were treated with increasing dose of SINE inhibitors. Proliferation of normal cells was not decreased as was observed with prostate cancer cells. Error bars represent mean ± SD (n=3). Asterisks over bars indicate significant (p<0.05, at least) statistical comparisons by the paired Student’s t test. Single asterisk indicates p<0.01, double asterisks indicate p<0.001, and triple asterisks indicate p<0.0001.


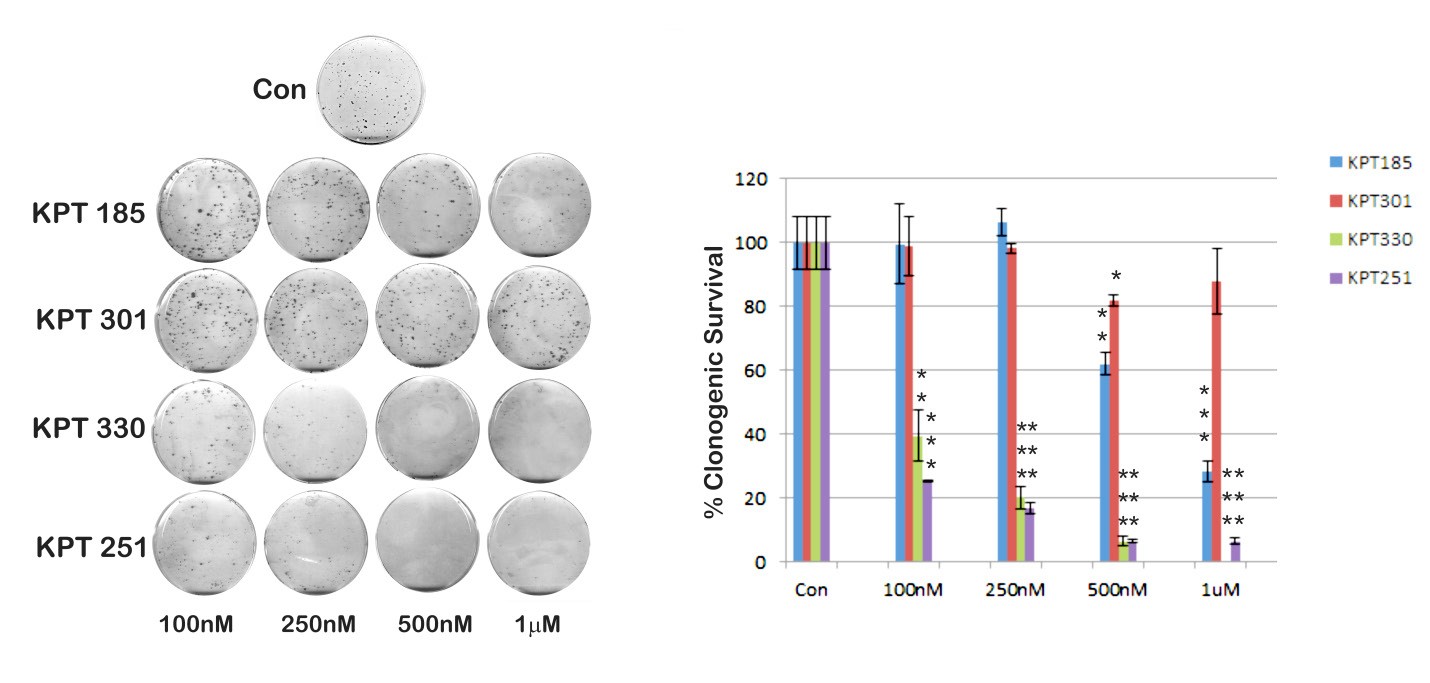


**Figure S3: Clonogenic analysis of PC3 cells treated with SINE inhibitors**. Graph represents quantitation of clonogenic survival. Error bar represent ± SD (n=3). Single asterisk indicates p<0.01, double asterisks indicate p<0.001, and triple asterisks indicate p<0.0001.
